# Air filtration and SARS-CoV-2

**DOI:** 10.4178/epih.e2020049

**Published:** 2020-07-04

**Authors:** Yevgen Nazarenko

**Affiliations:** Department of Atmospheric and Oceanic Sciences, McGill University, Montreal, QC, Canada

**Keywords:** Coronavirus, COVID-19, Aerosols, Filtration, HEPA, MPPS

## Abstract

Air filtration in various implementations has become a critical intervention in managing the spread of coronavirus disease 2019 (COVID-19). However, the proper deployment of air filtration has been hampered by an insufficient understanding of its principles. These misconceptions have led to uncertainty about the effectiveness of air filtration at arresting potentially infectious aerosol particles. A correct understanding of how air filtration works is critical for further decision-making regarding its use in managing the spread of COVID-19. The issue is significant because recent evidence has shown that severe acute respiratory syndrome coronavirus 2 (SARS-CoV-2) can remain airborne longer and travel farther than anticipated earlier in the COVID-19 pandemic, albeit with diminishing concentrations and viability. While SARS-CoV-2 virions are around 60-140 nm in diameter, larger respiratory droplets and air pollution particles (>1 µm) have been found to harbor the virions. Removing particles that could carry SARS-CoV-2 from the air is possible using air filtration, which relies on the natural or mechanical movement of air. Among various types of air filters, high-efficiency particle arrestance (HEPA) filters have been recommended. Other types of filters are less or more effective and, correspondingly, are easier or harder to move air through. The use of masks, respirators, air filtration modules, and other dedicated equipment is an essential intervention in the management of COVID-19 spread. It is critical to consider the mechanisms of air filtration and to understand how aerosol particles containing SARS-CoV-2 virions interact with filter materials to determine the best practices for the use of air filtration to reduce the spread of COVID-19.

Evidence is mounting that severe acute respiratory syndrome coronavirus 2 (SARS-CoV-2) can remain suspended in the air for extended periods. A fraction of airborne SARS-CoV-2 virions remains viable for at least 3 hours following aerosolization [1]. Polymerase chain reaction–positive SARS-CoV-2 was detected in aerosol particles larger than 1 µm in diameter in rooms accommodating patients with coronavirus disease 2019 (COVID-19) [2]. In another study, SARS-CoV-2 RNA was detected in the aerosol phase at distances of at least 3 m from infected people indoors [3]. SARS-CoV-2 RNA has also been found in air pollution particles traveling through the air [4].

The diameter of SARS-CoV-2 virions is around 60-140 nm [5]. Nonetheless, many exhaled respiratory droplets that can contain virions are substantially larger than the virions themselves. However, droplet evaporation in the air diminishes their size [6], allowing potentially infectious particles to remain airborne considerably longer. Dried droplets with a diameter of approximately 4 µm were observed to form from 12 µm to 21 µm speech-generated wet droplets due to drying [7]. These dried droplets took about 8 minutes to fall by only 30 cm in stagnant air [7]. At a low ambient temperature, highly humid exhaled breath can become supersaturated. Moisture then condenses on the particles emitted by a person, causing them to grow into droplets or ice crystals of a larger diameter. In such droplets or ice crystals, SARS-CoV-2 virions may stay viable longer, which is an important hypothesis that future research should test. Therefore, environmental conditions and aerosol dynamics can profoundly alter the wide range of exhaled particle sizes and the viability of SARS-CoV-2 virions in the aerosol particles mediating airborne transmission indoors and outdoors. The COVID-19 outbreaks at slaughterhouses and ski resorts may be at least in part due to the cold-air aerosol dynamics.

Removing particles that could harbor SARS-CoV-2 from the air using specialized air filtration equipment and using masks or respirators is an essential intervention in managing the COVID-19 spread. However, the effective deployment of air filtration has been hampered by a poor understanding of how air filtration works and misconceptions about the concept of filtration efficiency for aerosol particles of different sizes. It is critical to consider the mechanisms of air filtration and to understand how aerosol particles containing SARS-CoV-2 virions interact with filter materials to determine the best practices for the use of air filtration in managing the spread of COVID-19.

For air filtration, efficient particulate air (EPA) filters, high-efficiency particle arrestance (HEPA) filters, and ultra-low penetration air (ULPA) filters have been widely used in various industries and applications for many decades [8]. HEPA filters are recommended for infection control in healthcare settings [9,10] based on the balance of their high filtration efficiency and lower pressure drops compared to ULPA. HEPA filters are also deployed extensively in non-healthcare environments where airborne infectious agents may be present. Examples include the filtration of recirculated air in passenger aircraft and biosafety cabinets in laboratories, including those where SARS-CoV-2 research is conducted [11].

Often, the abbreviation HEPA is interpreted as “high-efficiency particulate air.” Both versions of the underlying term are used extensively, and there is no difference between them. The United States Department of Energy and the United States Environmental Protection Agency (EPA) define HEPA based on a minimum efficiency of 99.97% when tested with an aerosol of 0.3 µm diameter [12]. The United States EPA defines the diameter of 0.3 µm as the “most penetrating particle size” (MPPS). However, the MPPS can vary around 0.3 µm, with the precise value depending on the nature of the aerosol particles, the type of the filter material, and the flow rate [8]. Particles that are larger or smaller than MPPS are arrested with an efficiency higher than 99.97% [13]. The concept of MPPS is contrary to the widespread misconception that filtration efficiency drops for particles smaller than the MPPS (e.g., smaller than 0.3 µm). This misconception contributed to early policies that were misguided by the assumption that SARS-CoV-2 virions are too small to be effectively filtered out of the air.

HEPA filters are recommended to be installed on the outflow from the ventilators used in intensive care of people infected with SARS-CoV-2 [14]. Stationary (building ventilation) and portable HEPA filtration systems without and with air recirculation (indoor air purifiers) are recommended for use in healthcare settings by the United States Centers for Disease Control and Prevention and the World Health Organization, including in locations where SARS-CoV-2 patients are accommodated [10]. National and international standards govern the minimum filtration efficiency characteristics of HEPA filters. The two most commonly used standards are the international ISO 29463 standard and the European EN1822 standard [15]. The differences between the two standards are reconcilable [15]. For example, a HEPA filter certified to conform to EN 1822, filter class H14 standard must arrest at least 99.995% of aerosol particles at the MPPS. The EN 1822, filter class H14 standard is comparable to ISO 45 H [15]. Multi-step testing protocols are in place to verify the compliance of filters with the requirements of the standards [16,17]. When mechanical air movement across filters occurs, it may be important to ensure that no strong directional flows or drafts of filtered air are created. Concerns have recently been expressed that such directional flows could entrain unfiltered air that may contain infectious particles and push them faster and farther than they could spread in still air [18].

Antiviral properties can be imparted to the filter materials. However, once aerosol particles are collected on the filter fibers, virtually none detach and pass through the filter during or after proper use [8], so the antiviral properties of the fibers themselves have almost no effect on the removal of viable SARS-CoV-2 virions from the air. Particles that are deposited on previously collected particles do not contact the filter material, negating any antiviral properties. Therefore, imparting antiviral properties to HEPA-filter materials may not add value except where people come in direct contact with these filters during or shortly after use.

The mechanisms of aerosol particle filtration in the gas phase—inertial impaction, diffusion, interception, electrostatic deposition, and sieving [8,19]—have been investigated in depth over decades of research. These mechanisms have varying contributions to the total particle arrestance efficiency of filters depending on the particle aerodynamic diameter, other particle properties, and filtration media. The combined action of all these filtration mechanisms in HEPA filters explains their high efficiency of filtration across the entire aerosol size spectrum and the phenomenon of MPPS [8]. Various types of aerosol particles are filtered with high efficiency conforming to the corresponding standards, irrespective of their biogenic or non-biogenic origin [19].

It is known that some respiratory infections arise more frequently when people breathe more polluted air and that the recovery process and outcomes of certain respiratory infections are adversely affected by air pollution, based on numerous published studies. An association of higher COVID-19 mortality with the level of long-term particulate air pollution has already been shown [20]. Breathing polluted air is also strongly associated with adverse effects on respiratory and cardiovascular function [21]. Interventions based on air filtration using adequate equipment should be widely implemented both to reduce the spread of SARS-CoV-2 via the aerosol phase and to improve the health status and outcomes for people exposed to and infected with COVID-19.

## Ethics statement

This paper is a perspective, so it did not need ethical approval.
